# Intersectional race and gender disparities in kidney transplant access in the United States: a scoping review

**DOI:** 10.1186/s12882-023-03453-2

**Published:** 2024-01-25

**Authors:** Annika Gompers, Ana Rossi, Jessica L Harding

**Affiliations:** 1 Department of Epidemiology, Rollins School of Public Health, Emory University, 1518 Clifton Rd, Atlanta, GA 30322 USA; 2 Piedmont Transplant Institute, 1968 Peachtree Rd NW Building 77, Atlanta, GA 30309 USA; 3 Department of Surgery, Emory University School of Medicine, 100 Woodruff Circle, Atlanta, GA 30322 USA; 4 Health Services Research Center, Emory University School of Medicine, 100 Woodruff Circle, Atlanta, GA 30322 USA

**Keywords:** Kidney transplant, Gender, Race, Disparities, Transplant referral, Epidemiology, Health equity

## Abstract

**Background:**

Gender and racial disparities in kidney transplant access are well established, however how gender and race interact to shape access to kidney transplant is less clear. Therefore, we examined existing literature to assess what is known about the potential interaction of gender and race and the impact on access to kidney transplantation in the US.

**Methods:**

Following PRISMA guidelines, we conducted a scoping review and included quantitative and qualitative studies published in English between 1990 and May 31, 2023 among adult end-stage kidney disease patients in the US. All studies reported on access to specific transplant steps or perceived barriers to transplant access in gender and race subgroups, and the intersection between the two. We narratively synthesized findings across studies.

**Results:**

Fourteen studies met inclusion criteria and included outcomes of referral (*n* = 4, 29%), evaluation (*n* = 2, 14%), waitlisting (*n* = 4, 29%), transplantation (*n* = 5, 36%), provider perceptions of patient transplant candidacy (*n* = 3, 21%), and patient preferences and requests for a living donor (*n* = 5, 36%). Overall, we found that White men have the greatest access at all steps of the transplant process, from referral to eventual living or deceased donor transplantation. In contrast, women from racial or ethnic minorities tend to have the lowest access to kidney transplant, in particular living donor transplant, though this was not consistent across all studies.

**Conclusions:**

Examining how racism and sexism interact to shape kidney transplant access should be investigated in future research, in order to ultimately shape policies and interventions to improve equity.

**Supplementary Information:**

The online version contains supplementary material available at 10.1186/s12882-023-03453-2.

## Introduction

**Fig. 1:**
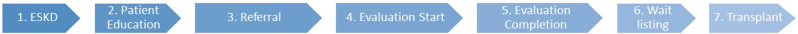
The six steps in kidney transplantation in the United States

Approximately 14%, or 31.2 million, of United States (US) adults are living with chronic kidney disease, of whom approximately 130,000 will progress to end-stage kidney disease (ESKD) each year [1]. For most of these patients, kidney transplantation is the superior treatment compared to dialysis, providing better quality of life, longer survival, lower costs, and lower hospitalization rates [2]. However, access to kidney transplant is not equitable for all ESKD patients. The transplantation process in the US is lengthy and complex, involving multiple health systems and including key steps of: patient education, referral for kidney transplantation, initiation of transplant evaluation, completion of evaluation, waitlisting, and transplantation (Fig. 1) [3]. Disparities by race [4, 5] and gender [6, 7] are well established [8], though how these groups intersect with one another to modify transplant access is underexplored.

Two recent reviews [6, 7] have summarized the extensive literature demonstrating that women are less likely than men to be referred for kidney transplant, complete evaluation, be placed on the transplant waitlist, and receive a deceased or living donor transplant. Women also generally wait longer to be transplanted once placed on the waitlist [9]. A plethora of evidence also documents the lower rates of referral, evaluation, waitlisting, and transplantation among ESKD patients of racial and ethnic minorities as compared to White ESKD patients, as well as longer waitlist times [4]. We have previously shown that gender disparities in referral for kidney transplantation vary by race in the Southeastern US [10]. Specifically, we have shown that race/ethnicity modified gender disparities such that White and Black women were 24% and 7% less likely to be referred than their male counterparts, respectively, and no statistically significant disparities were found among patients who were Hispanic or “other race.” How race and gender may interact with each other to differentially impact transplant access outside of the Southeast and across the whole transplant care continuum is unclear.

Health equity research is increasingly incorporating intersectionality, an analytical framework for understanding how multiple systems of oppression such as racism and sexism overlap and intersect to produce social inequalities [11]. Intersectionality has important applications to public health; studying health disparities by a single social factor masks variation within groups and prevents an understanding of how multiple individual-level social identities interact with multiple structural-level systems of power to jointly impact health outcomes [12]. Awareness of the drivers of health inequities and which subpopulations are most at risk is necessary in order to effectively develop and target interventions, rather than implementing a broad effort to increase access to kidney transplantation among all women or all Black patients, for example.

Therefore, in this scoping review we summarize the existing literature that has simultaneously examined gender and racial disparities in kidney transplant access in the US.

## Methods

This review adheres to the Preferring Reporting Items for Systematic Review and Meta-Analysis (PRISMA) extension for scoping reviews (PRISMA-ScR) guidelines (Table S1). As it is a review of existing studies, this study is considered exempt by the Emory University Institutional Review Board.

### Definitions

“Sex” refers to biological characteristics linked to sexual reproduction in males and females, including genetics, gonads, and genitals, and typically assigned at birth based on visual assessment of anatomy. “Gender” refers to the social and cultural norms, roles, behaviors, and interactions among people at the individual and societal level [13]. To date, most studies examining population-level disparities in kidney transplant are not designed to tease apart the effects of sex and gender. The majority of studies utilize only the variable “sex” as documented in medical records or ESKD registries, and do not collect data on gender identity. Moreover, sex and gender often cannot be easily disentangled, and use of the term “gender/sex” may sometimes be most appropriate [14]. In this review, we use “sex” to refer to the findings of studies, to reflect the label that exists in the underlying datasets and the terminology used in most of the articles included. We use the term “gender” when interpreting and synthesizing findings, to reflect the gendered social factors that likely underlie observed disparities between groups categorized by sex. We also use the term “race” to refer to socially constructed groups, often based on physical appearance, that serve as a proxy for experiences of racism [15].

### Search strategy

A literature search was conducted on May 31, 2023 to identify research articles published from 1990 onwards. Using a combination of text words related to gender, sex, race, ESKD and kidney transplantation, searches were performed in PubMed and Embase (Table S2). Results were filtered to include only studies on human adults and published in English. The reference lists of included studies were also screened for eligible articles.

### Eligibility criteria

We included full-text, original research articles publishing results from quantitative or qualitative studies conducted in the United States among patients with ESKD. Studies were required to report findings on access to kidney transplantation by sex and race simultaneously. Outcomes related to kidney transplant access that we included were referral, evaluation, waitlisting, and transplantation. We also included outcomes of patient or provider perceptions of transplantation, which may encompass barriers at multiple steps along the transplant process. Studies that considered sex and race as exposures in separate models were not included. However, we did include studies that assessed the effect of race within one sex category or the effect of sex within one racial category. Studies were excluded if the study population was patients with chronic kidney disease (rather than ESKD) or patients who had already received a transplant.

### Article selection

In order to screen articles, we entered all articles identified from PubMed and Embase into Covidence, an online systematic review management program. Each article’s title and abstract were screened by two reviewers (AG, JLH), and conflicts were resolved through discussion until the two reviewers reached consensus. Studies subsequently included in the full-text screen were screened by the same two reviewers, with the same process for resolving conflicts.

### Data analysis

One author (AG) extracted the following data from each included article: author names, title, year of publication, study design, study year, sample size, sample description (e.g. eligibility criteria), outcome, method used for intersectional analysis, and findings including effect size of crude and adjusted analyses where appropriate. However, we acknowledge that adjusted models may control for factors that lie on the causal pathway both between sex and the outcome, and race and the outcome of interest. For studies that did not report a measure of association (e.g. odds ratio or risk ratio) but provided frequencies or proportions of individuals with the outcome in each group, we calculated the measure of association using the data available and present this in Table 1. We have noted where we have performed these calculations and from which source table the data were extracted. We categorized the intersectionality methods utilizing a modified approach developed by Guan et al. [16] (Table S2). Categories of intersectionality methods include: (1) regression with interaction terms, (2) models using stratification, (3) approaches using categorized intersectional position, (4) methods to estimate mediation of intersectional effects, (5) prediction methods, (6) decomposition of inequality measures, (7) surrogate measures of additive interaction, (8) block/set regression, (9) presentation of raw data by race-gender group without formal statistical comparisons. Due to the small number of studies and heterogeneity in sample characteristics and outcomes, we used a qualitative approach to narratively synthesize the data and present findings by outcome.

**Table 1:** Summary of studies included in scoping review

First author (publication year)	Study year	Population, setting	Sample size	Outcome	Method category^1^	Unadjusted findings	Adjusted findings
Soucie (1992)	1989–1990	Black and White patients on dialysis; North Carolina, South Carolina, Georgia	8,315 (53% female, 32% White, 68% Black)	Transplant candidacy	Categorized intersectional position	**Unadjusted candidacy rates** White men: 16.7% White women: 13.7% Black men: 18.4% Black women: 13.1%	**Adjusted odds ratios** ^2^ White men: referent White women: 0.88 (95% CI 0.65–1.18) Black men: 0.77 (0.59–0.99) Black women: 0.66 (0.51–0.87)
Ojo (1993)	1983–1990	Black and White ESKD patients; United States	Not reported	Living related donor (LRD) transplantation rate	Stratification	**Unadjusted (transplantation rates)** *1983* White men 4x higher transplantation rate than Black men White women 4x Black women Black men 1.2x Black women White men 1.34x White women *1990* White men 5x Black men White women 4x Black women Black men 1x Black women White men 1.2x White women	NR
Narva (1996)	1990	Native American and White patients on dialysis or received a transplant; Arizona and New Mexico	8,851 (46% female, 18% Native American, 82% White)	Transplantation rate	Raw data	**Unadjusted risk ratios** ^3^ *Arizona* Native American men: referent White men: 1.06 (0.85–1.32) Native American women: referent White women 1.90 (1.39–2.61) *New Mexico* Native American men: referent White men 1.53 (1.09–2.14) Native American women: referent White women: 1.96 (1.32–2.89)	NR
McCauley (1997)	1990–1992	Black and White women initiating dialysis; Pennsylvania	276 (38% White, 62% Black)	Referral for transplantation; transplantation	Stratification (intra-categorical analysis)	**Unadjusted risk ratios** ^4^ *Referral* Black women: referent White women 0.98 (0.79–1.23) *Transplantation* Black women: referent White women 1.60 (1.04–2.48) *Time to referral* White women: 1.37 ± 0.24 years vs. Black women: 2.19 ± 0.3 years (*p* = 0.001)	**Adjusted risk ratios** ^5^ *Referral* Race not associated with referral (data not shown) *Transplantation* Black women: referent White women: 2.2 (95% CI 1.3-4.0)
Ayanian (1999)	1996–1997	Black and White patients initiating dialysis; Alabama, California, Michigan, Maryland, Virginia, Washington DC	1,392 (53% female, 48% White, 52% Black)	Referral; waitlisting or transplantation within 18 months of dialysis initiation; preference for kidney transplantation	Categorized intersectional position	**Unadjusted probability of the outcome** *Referral* White men: 82.3% vs. Black men: 60.4% (*p* < 0.001) White women: 75.2% vs. Black women: 55.5% (*p* < 0.001) *Waitlisting or transplantation* White men: 70.8% vs. Black men: 45.4% (*p* < 0.001) White women: 71.4% vs. Black women: 44.2% (*p* < 0.001) *Desire kidney transplant* White men: 85.5% vs. Black men: 80.7% (*p* = 0.04) White women: 79.3% vs. Black women: 76.3% (*p* = 0.13)	**Adjusted probability of the outcome** ^6^ *Referral* White men: 78.2% (referent) White women: 75.1% (95% CI 65.4–82.9) Black men: 61.2% (49.8–71.5) Black women: 59.9% (48.2–70.6) *Waitlisting or transplantation* White men: 62.7% (referent) White women: 64.7% (54.3–73.8) Black men: 48.7% (37.9–59.6) Black women: 44.6% (33.7–55.8)
Epstein (2000)	1996–1997	Black and White patients initiating dialysis; Alabama, California, Michigan, Maryland, Virginia, Washington DC	1,518 (52% female, 48% White, 52% Black)	Referral^7^; waitlisting; transplantation	Stratification	**Unadjusted** *Referral – appropriate candidates* White men: 98.8% vs. Black men: 86.5% (*p* = 0.005) White women: 97.2% vs. Black women: 94.1% (*p* = 0.43) *Referral – inappropriate candidates* White men: 62.7% vs. Black men: 36.6% (*p* < 0.001) White women: 53.9% vs. Black women: 40.0% (*p* = 0.02) *Waitlisting – appropriate candidates* White men: 90.3% vs. Black men: 61.1% (*p* < 0.001) White women: 82.5% vs. Black women: 81.8% (*p* = 0.93) *Waitlisting – inappropriate candidates* White men: 33.0% vs. Black men: 19.3% (*p* = 0.01) White women: 29.2% vs. Black women: 15.8% (*p* = 0.005) *Transplantation – appropriate candidates* White men: 58.8% vs. Black men: 16.2% (*p* < 0.001) White women: 44.4% vs. Black women: 17.7% (*p* = 0.007) *Transplantation – inappropriate candidates* White men: 10.8% vs. Black men: 3.4% (*p* = 0.02) White women: 9.9% vs. Black women: 1.2% (*p* < 0.001)	**Adjusted** ^8^ *Referral – appropriate candidates* White men: 96.0% vs. Black men: 90.9% (*p* = 0.57) White women: 96.1% vs. Black women: 93.1% (*p* = 0.60) *Referral – inappropriate candidates* White men: 67.7% vs. Black men: 36.4% (*p* = 0.013) White women: 58.8% vs. Black women: 43.1% (*p* = 0.21) *Waitlisting – appropriate candidates* White men: 85.9% vs. Black men: 68.7% (*p* = 0.12) White women: 82.4% vs. Black women: 85.6% (*p* = 0.49) *Waitlisting – inappropriate candidates* White men: 37.8% vs. Black men: 20.9% (*p* = 0.20) White women: 34.7% vs. Black women: 19.1% (*p* = 0.07) *Transplantation – appropriate candidates* White men: 62.2% vs. Black men: 16.4% (*p* = 0.002) White women: 41.0% vs. Black women: 18.8% (*p* = 0.14) *Transplantation – inappropriate candidates* White men: 22.8% vs. Black men: 6.0% (*p* = 0.04) White women: 23.5% vs. Black women: 1.5% (*p* = 0.01)
Thamer (2001)	1997–1998	Nephrologists; United States	271	Recommendation for kidney transplantation	Categorized intersectional position	**Unadjusted odds ratios** White men: referent White women: 0.50 (CI 0.38–0.65) Black women: 0.84 (0.65–1.08) Asian men: 0.61 (0.45–0.79)	**Adjusted odds ratios** ^9^ White men: referent White women: 0.41 (0.21–0.79) Black women: 0.78 (0.53–1.16) Asian men: 0.46 (0.24–0.91) No statistically significant difference between White women and Black women (results not shown)
Klassen (2002)	1996–1997	Black and White patients eligible for kidney transplant; Maryland	114 (44% female, 29% White, 71% Black)	Waitlisting	Regression with interaction term; stratification	NR	**Adjusted** ^10^ “African American and White men were both more likely to be listed than female patients; the sex effect was consistent across racial groups, and there was not a significant interaction effect between race and sex” (data not shown).
Clark (2008)	1996–1997	Black and White patients initiating dialysis; Alabama, California, Michigan, Maryland, Virginia, Washington DC	742 (50% female, 50% White, 50% Black)	Preference for transplant; physician recommendation for transplant^11^	Stratification	**Unadjusted odds ratios** ^12^ *Preference for transplant* White men: referent vs. Black men: 0.42 (CI 0.22–0.80) White women: referent vs. Black women: 0.92 (0.54–1.58) *Recommendation for transplant* White men: referent vs. Black men: 0.55 (0.33–0.93) White women: referent vs. Black women: 0.58 (0.35–0.94)	NR
Weng (2010)	2000–2005	Patients evaluated for kidney transplant; New Jersey	1,617 (39% female, 74% “non-Black,” 26% Black)	Recruitment of living kidney donors; receipt of living donor kidney transplant	Regression with interaction term	NR	**Adjusted** ^13^ No statistically significant interactions between race and sex (results not shown)
Gillespie (2014)	2008–2009	Black patients with ESKD on chronic hemodialysis; Pennsylvania	101 (52% female)	Waitlisting; evaluation for transplant; views on transplantation	Stratification (intra-categorical analysis)	**Unadjusted odds ratio** ^14^ *Waitlisting* Black men: referent vs. Black women: 0.51 (CI 0.20–1.28) *Evaluation* Black men: referent vs. Black women: 0.35 (0.15–0.80) *Would accept LDKT* Black men: referent vs. Black women: 0.28 (0.09–0.88) *Would accept DDKT* Black men: referent vs. Black women: 0.11 (0.02–0.54)	**Adjusted odds ratio** ^15^ *Would accept LDKT* Black men: referent vs. Black women: 0.15 (CI 0.04–0.46)^16^
Monson (2015)	2009–2010	Patients presenting for initial kidney transplant evaluation; Illinois	256 (43% female, 22% White, 50% Black, 29% Hispanic)	Rate of completion of pre-transplant evaluation within 12 months of initial renal transplant clinic visit	Regression with interaction term; categorized intersectional position	NR	**Adjusted hazard ratios** ^17^ Black men: referent vs. Black women: 1.38 (*p* = 0.16) White men: 1.99 (*p* = 0.005) White women: 0.94 (*p* = 0.83) Hispanic men: 2.75 (*p* < 0.0001) Hispanic women: 1.96 (*p* = 0.006) Statistically significant interaction between race/ethnicity and sex on completion (*p* = 0.02) White women: referent vs. Black women: 1.80 (*p* = 0.08) Hispanic women: 2.18 (*p* = 0.02)
Gillespie (2020)	2012–2014	Patients waitlisted for kidney transplant; Virginia	128 (53% female, 32% White, 68% Black)	Number of living donor requests	Stratification	NR	**Adjusted incidence rate ratios** ^18^ White men: referent vs. White women: 3.06 (CI 1.43–6.55) Black men: referent vs. Black women: 1.62 (0.98–2.67)
Smothers (2022)	2012–2016	ESKD patients initiating dialysis; North Carolina, South Carolina, Georgia	45,015 (45% female, 42% White, 53% Black, 3% Hispanic, 2% “Other” race)	Referral	Regression with interaction term; stratification	NR	**Adjusted odds ratios** ^19^ White men: referent vs. White women: 0.76 (CI 0.71–0.82) Black men: referent vs. Black women: 0.93 (0.88–0.99) Hispanic men: referent vs. Hispanic women: 0.85 (0.65–1.12) “Other race” men: referent vs. “Other race” women: 0.78 (0.56–1.09) There was a statistically significant interaction between race and sex (*p* = 0.001)

NR = not reported. 95% CI = 95% confidence interval. LDKT = living donor kidney transplant. DDKT = deceased donor kidney transplant

^1^ Drawn from the categories in Guan et al. 2021

^2^ Adjusted for age, time on dialysis, cause of ESKD, functional and nutritional status, comorbid conditions, and socioeconomic factors

^3^ Calculated using data in Narva et al. Table 6

^4^ Calculated using data from text on McCauley et al. page 741 and 743

^5^ Model for referral was adjusted for age, education, employment, county of residence, and comorbidities. Model for transplantation was adjusted for age, education, employment, county, hospitalization, comorbidities, cause of ESKD, and dialysis modality

^6^ Adjusted for patient preferences, sociodemographic factors, type of dialysis facility, perceptions of care, health status, cause of kidney failure, and coexisting conditions

^7^ Referral was assessed by chart review and through patient survey separately. This table presents only the results from referral ascertained via chart review

^8^ Adjusted for age, region, cause of ESKD, education, income, health status, preferences, and distance to transplant facility

^9^ Adjusted for age, compliance with treatment, weight, residual renal function, cardiac ejection fraction, HIV status, living arrangement, and nephrologist characteristics

^10^ Adjusted for age, sex, employment, years with ESKD, previous treatment, self-rated health, attitude toward transplantation, experience with discrimination

^11^ The main exposure of interest in this study was level of social support, the main outcome was completion of evaluation, and the authors assessed the association separately in each race-gender group (Clark et al. Table 2). For the purposes of this review, we focus on the outcomes of preference and recommendation for transplant, which are presented stratified by race-gender group in Clark et al. Table 1

^12^ Calculated using data from Clark et al. Table 1

^13^ Adjusted for age, marital status, dialysis status, insurance, panel reactive antibodies, and blood type

^14^ Calculated using data from Gillespie et al. Table 3, excluding “Do not know” responses

^15^ Adjusted for age, marital status, education, insurance, peripheral vascular disease, and survey administration mode

^16^ Results from best internal validation full-sample model. Results from best casewise-deleted subsample model and base casewise-deleted full-sample model were similar

^17^ Adjusted for race/ethnicity, insurance, marital status, time on dialysis, hospitalizations, previous incomplete workup, in need of stress test, lower extremity arterial study, carotid duplex, colonoscopy or Pap test, total number of tests needed

^18^ Adjusted for age, marital status, income, and transplant knowledge

^19^ Adjusted for age, BMI, insurance, primary cause of ESKD, pre-ESKD care, transplant education, and comorbidities. Dialysis facility modeled as a random effect

## Results

**Fig. 2:**
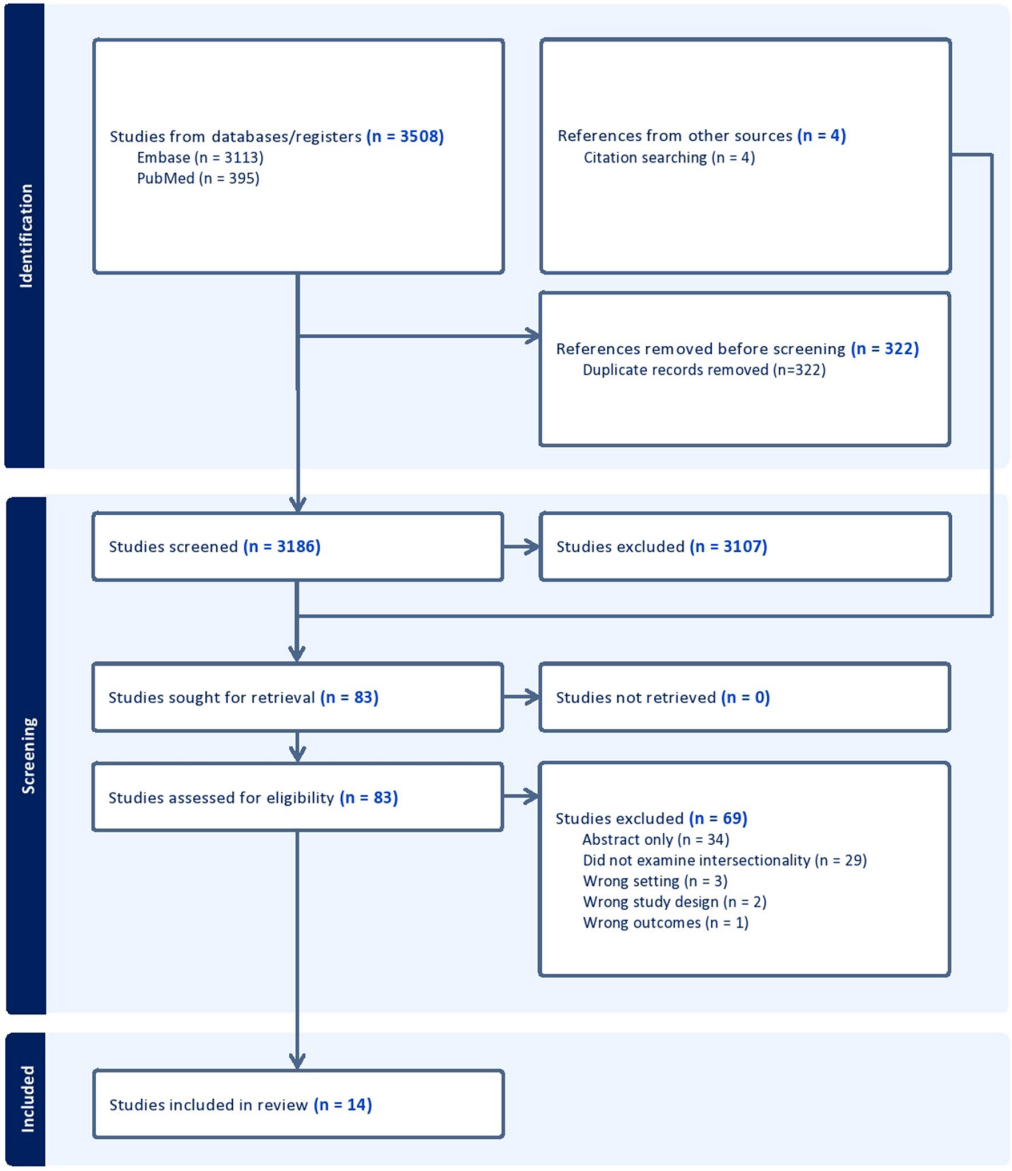
PRISMA flow diagram of literature search for scoping review

The PubMed search yielded 395 results, and the Embase search yielded 3,113. An additional four articles were identified from reference list screening. After removing 322 duplicates, 3,186 studies were screened in Covidence. After title and abstract screening, 83 studies were included for full text screening. Of these, 69 were excluded for being an abstract rather than full text article (49%), not examining race and sex simultaneously (42%), or having the wrong setting (4%), wrong study design (3%), or wrong outcome (1%). Fourteen articles were included in the final sample (Fig. 2).

The characteristics of each of the 14 included articles are summarized in Table 1. All of the 14 articles were quantitative analyses, though one also included a qualitative component [17]. Seven studies conducted retrospective reviews of medical records or registry data, two were survey studies, and five included both a retrospective record review and a survey. Eight studies (57%) were published between 1992 and 2002, and six (43%) published between 2008 and 2022. The articles reported on data covering a wide area of the US, including the Northeast (New Jersey), Mid-Atlantic (Pennsylvania, Maryland, Virginia, Washington DC), Southeast (North Carolina, South Carolina, Georgia, Alabama), Midwest (Illinois, Michigan), Southwest (Arizona, New Mexico) and West (California). There were two national studies [18, 21]. Approaches to intersectional analysis varied, with four studies (29%) using regression methods with interaction terms [10, 17, 19, 20], four studies (29%) categorizing intersectional position into a single variable [20–23], eight studies (57%) conducting stratified analyses [10, 17, 18, 24–27, 29,], and two studies (14%) presenting raw statistics for each race-gender group separately [18, 28]; three studies (21%) implemented more than one approach.

### Referral

Four studies (29%) examined the outcome of referral. In a cohort of 1,392 patients initiating dialysis in Alabama, California, Michigan, Maryland, Virginia, or Washington, DC, Ayanian et al. found that the proportion of patients referred was 55.5% among Black women, 60.4% among Black men, 75.2% among White women, and 82.3% among White men [22]. In multivariable models adjusting for patient preferences, sociodemographic factors, and medical conditions, Black women and Black men – but not White women – had a lower probability of referral compared to the referent of White men. In a study of 45,015 patients initiating dialysis in the Southeastern US, women overall were 14% less likely to be referred than men, with a statistically significant interaction between race and sex (*p* = 0.001) [10]. White women were 24% less likely to be referred than White men, Black women were 7% less likely to be referred than Black men, and there were no statistically significant sex disparities among Hispanic patients or patients of another race.

In a study of 276 women initiating dialysis in Pennsylvania, White women and Black women were referred at similar rates (53.8% and 54.7%, respectively), though no comparison was made to men [27]. In a cohort of 1,518 Black and White patients overlapping with the cohort analyzed by Ayanian et al. [22], Epstein et al. differentiated between “appropriate” and “inappropriate” candidates for transplantation based on the number and severity of contraindications to kidney transplant [26]. Appropriate candidates had no contraindications and inappropriate candidates had at least one absolute contraindication (such as active malignant condition or HIV infection) or at least three relative contraindications (such as body mass index of 30 to 35 or moderate coronary artery disease). After adjustment for age, education, income, health status, patient preferences, and geography, there were no statistically significant differences in referral rates by race among appropriate male candidates (96.0% of White men vs. 90.9% of Black men, *p* = 0.57), appropriate female candidates (96.1% of White women vs. 93.1% of Black women, *p* = 0.60), or inappropriate female candidates (58.8% of white women vs. 43.1% of Black women, *p* = 0.21). However, among inappropriate male candidates, White men were more likely to be referred (67.7%) than Black men (36.4%, *p* = 0.01).

### Evaluation

Two studies (14%) assessed completion of the evaluation process by sex and race. A study of 101 Black patients on chronic hemodialysis in Philadelphia found that Black men were more likely to be evaluated for transplant than Black women in unadjusted models (52.2% vs. 28.3%, *p* = 0.01) [25]. Meanwhile, a study of 256 patients presenting for initial kidney transplant evaluation in Chicago did not find a statistically significant difference in rate of completion of evaluation between Black men and women (HR 1.38, *p* = 0.16) in multivariable models [20]. However, compared to the reference of Black men, there was faster completion among Hispanic men (HR 2.75, *p* < 0.001), Hispanic women (HR 1.96, *p* = 0.006), and White men (HR 1.99, *p* = 0.005), and faster completion among Hispanic women compared to White women (HR 2.18, *p* = 0.02). Overall, there was statistically significant interaction between race and sex on completion of evaluation (*p* = 0.02).

### Waitlisting

Three studies (21%) examined waitlisting as the outcome and one study (7%) examined the combined outcome of waitlisting and transplant and is described here. In a cohort of 114 transplant-eligible patients on dialysis in Maryland, men were more likely than women to be waitlisted among both Black and White patients, after adjusting for demographics, medical history, attitude towards transplant and experiences with discrimination (effect size was not reported) [17]. This sex disparity was consistent across races, and no statistically significant interaction between race and sex was reported. Qualitative interviews with this cohort indicated that overall, patients experienced little discrimination in their dialysis units, however Black patients reported experiencing more discrimination in unfamiliar care settings; this could act as a barrier to waitlisting among Black patients, who may feel hesitant to seek care in new settings such as transplant clinics [17]. In another cohort of 101 Black ESKD patients, Black men were waitlisted at a higher rate than Black women (31.3% vs. 18.9%), though this difference was not statistically significant (*p* = 0.15) [25]. Epstein et al. reported that in crude analyses, White patients were more likely to be waitlisted than Black patients among both men and women considered to be “appropriate” transplant candidates (see earlier description), and among men considered to be “appropriate” candidates [26]. After adjustment, however, race was not associated with likelihood of waitlisting among any group of patients (i.e., appropriate and inappropriate men and women).

Among 1,115 patients initiating dialysis in Alabama, California, Michigan, Maryland, Virginia, or Washington, DC who reported definitely wanting a kidney transplant, White patients were more likely to be waitlisted or transplanted within 18 months compared to Black patients among both women and men (*p* < 0.01 for both); 44.2% of Black women, 71.4% of White women, 45.4% of Black men and 70.8% of White men [22]. After adjusting for patient preferences, sociodemographic factors, type of dialysis facility, and health status, the probability of waitlisting or transplantation was still lower among Black women (59.9%) and Black men (61.2%) compared to White men (78.2%), but not among White women (75.1%) compared to White men. Of note, this study excluded patients who reported not wanting or being unsure if they wanted a kidney transplant, which is known to differ by race and sex, with 76.3% of Black women, 79.3% of White women, 80.7% of Black men, and 85.5% of White men reporting wanting to receive a kidney transplant [22].

### Transplantation

Five studies (36%) reported on disparities in kidney transplantation. In a national study of ESKD patients on dialysis between 1983 and 1989, racial disparities were noted among both men and women: White men had a five times higher rate of living related donor (LRD) transplant than Black men, and White women had a four times higher rate of LRD than Black women [18]. The same study found that Black and White men received LRD transplants at higher rates than their female counterparts in 1983 (20% and 34% higher, respectively), though by 1990 these disparities disappeared among Black patients and decreased from 34 to 20% among White patients. Similarly, in a cohort of 8,851 ESKD patients in the Southwestern US, transplantation rates were higher among men than women and higher among White patients than Native American patients, with White men having the highest rates and Native American women having the lowest [28]. In New Mexico, for example, 20.9% of White men, 17.0% of White women, 13.7% of Native American men, and 8.7% of Native American women with ESKD were transplanted. Figures were similar in Arizona.

In a study of 276 female patients initiating dialysis in Pennsylvania, White women were 2.2 times as likely to receive a transplant than Black women (95% CI 1.3-4.0), after adjusting for age, educational status, employment status, county of residence, hospitalization, comorbidities, cause of ESKD, and dialysis modality [27]. Time to transplantation was also shorter in White women than Black women (1.37 ± 0.24 years vs. 2.19 ± 0.30 years, *p* = 0.001) in unadjusted analyses. In their study of 1,518 Black and White patients initiating dialysis, Epstein et al. similarly adjusted for age, education, income, cause of ESKD, health status, patient preferences and geography, and found that a higher proportion of White men than Black men were transplanted among both appropriate candidates (i.e. no contraindications to transplant; 62.% of White men vs. 16.5% of Black men, *p* = 0.002) and inappropriate candidates (22.8% of White men vs. 6.0% of Black men, *p* = 0.04) [26]. Among women, the difference in transplantation rates by race was only statistically significant among inappropriate candidates (23.5% of White women vs. 1.5% of Black women, *p* = 0.01), not appropriate candidates (41.0% of White women vs. 18.8% of Black women, *p* = 0.14). In a study of 1,617 patients being evaluated for a kidney transplant in New Jersey, Weng et al. found no statistically significant interaction between race and sex in models adjusted for demographic and medical variables [19].

### Provider perception of patient transplant candidacy

Three studies (21%) focused on provider reports of patients’ candidacy for kidney transplantation. One assessed the transplant candidacy status (classified as yes or no) of 8,315 patients in the Southeastern US as reported by the patient’s dialysis center [23]. In that population, using White men as the referent group and adjusting for age, time on dialysis, cause of ESKD, health status, and socioeconomic factors, the likelihood of being identified as a transplant candidate was lower among Black men (Odds Ratio (OR): 0.77 [95% CI 0.59–0.99]) and Black women (OR: 0.66 [0.51–0.87]), but not different for White women (OR: 0.88 [0.65–1.18]). In a survey of 742 patients initiating dialysis, patients were asked whether a physician had recommended the option of transplantation to them. Overall, 80% percent of White men and 69% of Black men had been recommended transplantation (*p* = 0.03), and 78% of White women and 67% of Black women had (*p* = 0.02) [29]. In a national survey of nephrologists, participants were presented with hypothetical scenarios of patients initiating dialysis and asked to decide whether to recommend each “patient” for transplantation [21]. Using White men as the referent group and adjusting for age, living arrangement, compliance with treatment, health indicators, and provider demographics, there were lower odds of recommendation for transplantation for White women (OR: 0.41 [0.21–0.79]) and Asian men (OR: 0.46 [0.24–0.9]), but not Black women (OR: 0.78 [0.53–1.16])

### Patient perception of transplantation and requests for a living donor

Five studies (36%) investigated sex and racial disparities in patients’ perceptions of kidney transplantation or requests for a living donor. Among a sample of 1,392 patients initiating dialysis, similar proportions of White women (79.3%) and Black women (76.3%) reported wanting a kidney transplant (*p* = 0.13), and a higher proportion of White men (85.5%) than Black men (80.7%) desired a transplant (*p* = 0.04) [22]. In a later analysis of a subset of the same cohort, restricted to 742 patients without critical contraindications to transplantation, results were unchanged; desire for kidney transplant was still similar among White and Black women (83% vs. 82%, *p* = 0.77), and still higher among White men than Black men (91% vs. 82%, *p* = 0.01) [29]. In a survey of 101 Black ESKD patients, men reported more willingness to accept a transplant than women, with 87.5% of men willing to accept a living donor kidney transplant and 85.4% of men willing to accept a deceased donor transplant, compared to only 52.5% and 56.6% of women, respectively [25]. After adjusting for age, marital status, education, insurance type, peripheral vascular disease, and survey administration mode, Black women were approximately 85% less likely to report wanting a living donor kidney transplant than Black men (OR: 0.15 [0.04–0.46] for best internal validation full-sample model). In a separate survey of 128 Black and White waitlisted patients, Black women made a similar number of requests for a living donor as Black men (Incidence Rate Ratio (IRR): 1.62, [95% CI 0.98–2.67]), but White women made more requests than White men (IRR: 3.06 [1.43–6.55]) [24]. In a cohort of 1,617 patients being evaluated for a kidney transplant in New Jersey, there was no statistically significant interaction between race and sex on recruitment of potential living donors (results were not shown) [19].

## Discussion

Despite well-known disparities in transplant access by race and gender, in this scoping review, we found just 14 studies – and just two published after 2015 – that investigate the intersectionality between the two. It is surprising that this body of literature remains small, even though joint gender and racial disparities in kidney transplant have been reported for more than 30 years [30]. A recent review has also identified that the rate of research on (separate) racial inequities and gender inequities in kidney transplantation has appeared to stagnate [8]. This is despite the rapidly growing field of health equity research [31, 32] and the recent increased attention to the importance of examining intersectionality in public health [12]. Among the limited existing evidence base, we find that White men have the greatest access at all steps of the transplant process, from referral to eventual living or deceased donor transplantation. In contrast, women from racial or ethnic minorities tend to have the lowest access to kidney transplant, in particular living donor transplant, though this was not consistent across all studies. For example, in a national survey of nephrologists, when asked to recommend hypothetical patients for transplant, Black women were similarly likely to be recommended for transplant compared to White men and White women [21]. In a study of waitlisted patients in Virginia, White women made more requests for a living donor than White men while Black women made a similar number of requests as Black men [24]. There were also null findings of interactions between race and gender on the outcomes of waitlisting in Maryland [17] and transplantation in New Jersey [19]. This study highlights the importance of examining intersectionality by race and gender, two well-known factors affecting access to kidney transplantation that are typically studied independently, but which can interact together to modify transplant access. Given the sparse literature to date, intersectional analyses should be an essential component of future studies aiming to understand and eventually enable the design of effective policies to address inequities in access to care.

Systems of power and discrimination such as racism and sexism can drive health disparities through many mechanisms, including economic deprivation, social deprivation, and restricted medical care [33, 34]. As articulated through the existing framework of intersectionality, individuals hold multiple identities (e.g., race and gender) that intersect with these macro-level power systems, and as such, a person’s physical, mental, and material wellbeing are best understood in the context of the multiple structural factors being experienced [12]. While none of the studies included in this review explicitly referred to intersectionality, they do illustrate an understanding that multiple social identities (and by proxy, systems of power and oppression) intersect and influence health. As such, similar future research may be strengthened by using language explicitly referring to intersectionality, along with appropriate definitions, references to relevant literature, and discussion of potential mechanisms at play.

In the case of kidney transplantation, disparities are likely driven by sexism, racism, and their interaction. Though pregnancy-induced sensitization is often considered a biological sex-related barrier to transplant for women, participation in a kidney paired exchange program has shown that sex-based differences in living donor kidney transplant can be eliminated, illustrating that enacting appropriate policies can achieve gender/sex equity in living donor transplant access despite any biological differences [35]. Further, the impact of sex on conditions related to suitability for transplantation, such as frailty and obesity, are likely also entangled with gender [36]; while biological factors appear to contribute to sex differences in frailty, these differences are likely inflated by the fact that women are often more willing to identify and report health issues [37], and healthcare providers tend to perceive women as frailer than men [38]. Physician bias likely also interacts with patient obesity, as obesity has a greater impact on waitlisting [39] and transplantation [40] rates among women than among men. Additional gendered factors include caregiving burden, which disproportionality impacts women and can create barriers for seeking timely care [6, 41]. Despite being less likely to receive or accept a live donor kidney, women are more likely to be living donors, particularly in the context of a heterosexual marriage [42] or family, in which gender roles (including caregiving and ability to take time off work) likely influence a woman’s decision to donate a kidney to her husband or family member [43, 44].

Systemic racism operates at multiple levels to influence kidney transplant access; one recent example is the finding that historical incorporation of a race correction in eGFR calculation causes delays in kidney transplant eligibility for Black ESKD patients [45]. Differential access to economic capital by both race and gender – due to differential educational and employment opportunities as well as generational wealth – likely drives transplant access; adequate economic resources are necessary for many steps of the transplant process, including time off work and transportation for the many medical visits, access to constant caregiving after transplantation, and the cost of immunosuppressant drugs after transplantation. Women and racial/ethnic minorities are also less likely to have adequate insurance coverage, an established barrier to transplant access [46–49]. Socioeconomic position also determines place of residence and therefore proximity to healthcare facilities. Beyond distance from a transplant center, dialysis facility-level factors such as for-profit status and lower patient to social worker ratio have been shown to pose barriers to kidney transplant referral [3, 50]. In addition, more direct experiences of discrimination likely influence transplant access along gendered and racial lines simultaneously. For example, providers may view patients who are women and/or people of color as less suitable for transplant or less of a priority [21]. Support for kidney transplantation among family or community members, including willingness to serve as a living donor, may also be lower for individuals with (multiple) marginalized identities [29, 44, 51]. Financial costs and concern over health risks of donation also serve as barriers to living kidney donation among Black populations [44].

Finally, racism and sexism are well-established upstream determinants of poor health [52–55], making it more likely that women and racial/ethnic minorities will develop obesity, cardiac disease, and other medical contraindications to transplant [56–58]. However, many studies adjust for these medical factors and still find racial and gender disparities [e.g. 10, 22, 23, 27], suggesting likely social causes of disparities, rather than absolute contraindications. At the same time, adjusting for clinical and demographic factors might mask causal mechanisms for how racism and sexism intersect to shape kidney transplant access. For example, Epstein et al. adjusted for age, region, cause of ESKD, education, income, health status, preferences, and distance to transplant facility [26], and Weng et al. adjusted for age, marital status, dialysis status, insurance, panel reactive antibodies, and blood type [19]. It is likely that variables such as education, income, and insurance lie on the causal pathway between race, gender and transplantation, with racism and sexism causing disparities in socioeconomic factors that impact transplant access. Adjusting for these structural disparities may limit studies to assessing the impact of interpersonal or internalized racism. Therefore, studies examining the intersection between race and sex should be thoughtful when selecting covariates, ideally by creating a directed acyclic graph to identify causal pathways. Additionally, including crude results can be important for understanding the net effects of racism and sexism.

This is the first scoping review of the literature studying the intersection of race and gender in disparities in kidney transplant access. We searched two databases using text word terms, supplemented by manual reference list screening, minimizing the chance of missing key studies. However, there are some limitations. First, we did not include a search of gray literature or reports. We also did not include studies on the late-stage CKD population, despite the optimal treatment being preemptive kidney transplant [59]. However, we believe that our inclusion criteria of a diagnosis of ESKD captures the majority of patients initiating the kidney transplant process as our previous work has suggested that only approximately 18% of all individuals being referred to a transplant center have late-stage CKD [48]. Furthermore, this review is limited to studies in the US and is therefore not generalizable to international contexts, though this was by design as the healthcare and kidney allocation system in the US is unique. In addition, comparing results across studies included in this review is difficult due to differences in study populations, years of data collection, outcomes, and intersectionality methods employed. There was also variation within a single analytic approach such as categorized intersectional position, with some studies selecting White men as the reference group [21–23] but others selecting Black men as the referent [20]. Stratification was also carried out to assess the effect of race within gender in some studies [26, 27, 29], but the effect of gender within race in others [10, 24, 25]. We were also limited to the intersectionality approaches employed by individual studies in this review, which do not include the more advanced methods available [16, 60]. Of note, however, is that intersectionality was developed as a theoretical framework and not a hypothesis that can be empirically tested [61]. As such, intersectional disparities and the variation across time, space, and outcome may be best investigated through qualitative methods, either alone or alongside quantitative methods. Of note, however, is that only one of the 14 studies we found that investigated the intersection between race and gender included a qualitative analysis [17], indicating the underutilization of qualitative methods in this area to date. Finally, we recognize that race and gender likely intersect with other identities including socioeconomic status, disability, sexual orientation, and immigration status, which were not considered in this scoping review.

## Conclusion

Access to kidney transplantation is shaped by both race and gender and the intersection between the two. In particular, women of racial/ethnic minorities tend to have the least access to each step of the complex transplant care continuum, especially living donor transplant. Although this evidence dates back several decades, the body of literature on intersectional disparities in kidney transplant access remains small. Therefore, a priority must be to expand research investigating how racism and sexism interact to shape kidney transplant access. Only then, with a better understanding of key mechanisms and processes, can policies and interventions be developed to address these inequities.

### Electronic supplementary material

Below is the link to the electronic supplementary material.

**Supplementary Material 1: Table S1.** Preferred reporting items for systematic reviews and meta-analyses extension for scoping reviews (PRISMA-ScR) checklist. **Table S2.** Key search terms used in PubMed search strategy. **Table S3.** Definition of categories of quantitative intersectionality approach, adapted from Guan et al. 2021

## Data Availability

No datasets were generated or analysed during the current study.

## Abbreviations

ESKD: End Stage Kidney Disease
LRD: Living Related Donor
US: United States
