# Supplementary material for: Intersectional race and gender disparities in kidney transplant access in the United States: a scoping review
Source: BMC Nephrol. 2024 Jan 25;25:36. doi: 10.1186/s12882-023-03453-2 (PMC10811805; doi:10.1186/s12882-023-03453-2)
Supplement: Supplementary file 1 — Supplementary Material 1: Table S1. Preferred reporting items for systematic reviews and meta-analyses extension for scoping reviews (PRISMA-ScR) checklist. Table S2. Key search terms used in PubMed search strategy. Table S3. Definition of categories of quantitative intersectionality approach, adapted from Guan et al. 2021 [file 12882_2023_3453_MOESM1_ESM.pdf]

Table S1: Preferred Reporting Items for Systematic Reviews and Meta-Analyses extension for Scoping Reviews (PRISMA-ScR) Checklist

SECTION	ITEM	PRISMA-ScR CHECKLIST ITEM	REPORTED ON PAGE #
**TITLE**			
Title	1	Identify the report as a scoping review.	Title page
**ABSTRACT**			
Structured summary	2	Provide a structured summary that includes (as applicable): background, objectives, eligibility criteria, sources of evidence, charting methods, results, and conclusions that relate to the review questions and objectives.	2
**INTRODUCTION**			
Rationale	3	Describe the rationale for the review in the context of what is already known. Explain why the review questions/objectives lend themselves to a scoping review approach.	3
Objectives	4	Provide an explicit statement of the questions and objectives being addressed with reference to their key elements (e.g., population or participants, concepts, and context) or other relevant key elements used to conceptualize the review questions and/or objectives.	4
**METHODS**			
Protocol and registration	5	Indicate whether a review protocol exists; state if and where it can be accessed (e.g., a Web address); and if available, provide registration information, including the registration number.	Not done
Eligibility criteria	6	Specify characteristics of the sources of evidence used as eligibility criteria (e.g., years considered, language, and publication status), and provide a rationale.	5
Information sources*	7	Describe all information sources in the search (e.g., databases with dates of coverage and contact with authors to identify additional sources), as well as the date the most recent search was executed.	5
Search	8	Present the full electronic search strategy for at least 1 database, including any limits used, such that it could be repeated.	Table S2
Selection of sources of evidence†	9	State the process for selecting sources of evidence (i.e., screening and eligibility) included in the scoping review.	5
Data charting process‡	10	Describe the methods of charting data from the included sources of evidence (e.g., calibrated forms or forms that have been tested by the team before their use, and whether data charting was done independently or in duplicate) and any processes for obtaining and confirming data from investigators.	6
Data items	11	List and define all variables for which data were sought and any assumptions and simplifications made.	6
Critical appraisal of individual sources of evidence§	12	If done, provide a rationale for conducting a critical appraisal of included sources of evidence; describe the methods used and how this information was used in any data synthesis (if appropriate).	Not done
Synthesis of results	13	Describe the methods of handling and summarizing the data that were charted.	6
**RESULTS**			

SECTION	ITEM	PRISMA-ScR CHECKLIST ITEM	REPORTED ON PAGE #
Selection of sources of evidence	14	Give numbers of sources of evidence screened, assessed for eligibility, and included in the review, with reasons for exclusions at each stage, ideally using a flow diagram.	6, Figure 1
Characteristics of sources of evidence	15	For each source of evidence, present characteristics for which data were charted and provide the citations.	7, Table 1
Critical appraisal within sources of evidence	16	If done, present data on critical appraisal of included sources of evidence (see item 12).	Not done
Results of individual sources of evidence	17	For each included source of evidence, present the relevant data that were charted that relate to the review questions and objectives.	7-12, Table 1
Synthesis of results	18	Summarize and/or present the charting results as they relate to the review questions and objectives.	6-12
**DISCUSSION**			
Summary of evidence	19	Summarize the main results (including an overview of concepts, themes, and types of evidence available), link to the review questions and objectives, and consider the relevance to key groups.	12-13
Limitations	20	Discuss the limitations of the scoping review process.	15
Conclusions	21	Provide a general interpretation of the results with respect to the review questions and objectives, as well as potential implications and/or next steps.	13-14
**FUNDING**			
Funding	22	Describe sources of funding for the included sources of evidence, as well as sources of funding for the scoping review. Describe the role of the funders of the scoping review.	17

From: Tricco AC, Lillie E, Zarin W, O'Brien KK, Colquhoun H, Levac D, et al. PRISMA Extension for Scoping Reviews (PRISMAScR): Checklist and Explanation. Ann Intern Med. 2018;169:467–473. doi: 10.7326/M18-0850.

Table S2: Key search terms used in PubMed search strategy

Category*	Search Term	Operator
Gender/sex	Gender<br>Sex	OR
Race/ethnicity	Race<br>Ethnicity<br>Black<br>White<br>Hispanic<br>Asian	OR
ESKD	End-stage kidney disease<br>Kidney failure<br>Chronic kidney disease	OR
Transplant access	Kidney transplantation<br>Waitlist*<br>Waiting list<br>Referral<br>Evaluation<br>Living donor<br>Deceased donor	OR
Broader search term	(Gender[Text Word] OR sex[Text Word]) AND (race[Text Word] OR ethnicity[Text Word] OR Black[Text Word] OR White[Text Word] OR Hispanic[Text Word] OR Asian[Text Word]) AND (end-stage kidney disease[Text Word] OR kidney failure[Text Word] OR chronic kidney disease[Text Word]) AND (kidney transplantation[Text Word] OR waitlist*[Text Word] OR waiting list[Text Word] OR referral[Text Word] OR evaluation[Text Word] OR living donor[Text Word] OR deceased donor[Text Word])<br><br>Filters: Humans, English, Adult: 19+ years, Young Adult: 19-24 years, Adult: 19-44 years, Middle Aged + Aged: 45+ years, Middle Aged: 45-64 years, Aged: 65+ years, 80 and over: 80+ years, from 1990 - 3000/12/12	

* All categories combined with ‘AND’ operator

Table S3: Definition of categories of quantitative intersectionality approach, adapted from Guan et al. 2021

**Approach**	**Definition**
Regression with interaction terms	Using multivariable regression models that include an interaction term, e.g. interaction between race and gender/sex
Stratification	Stratifying regression models to estimate effect modification, e.g. if the effect of race differs by gender/sex strata, or if the effect of gender/sex differs by race strata
Categorized intersectional position	Categorizing intersectional position using a single variable, e.g. with the levels White men, White women, Black men, Black women, and comparing outcomes against a reference group (e.g. White men)
Estimation of mediation of intersectional effects	Investigating whether mediators such as discrimination explain outcomes across intersectional positions using methods such as simple mediation analysis and structural equation modeling
Prediction methods	Identifying interactions between intersectional positions that best predict the outcome
Decomposition of inequality measures	Decomposing inequality in the outcome into components that can be attributed to each individual social position (e.g. Black and female) and to the intersectional position (Black female)
Surrogate measures of additive interaction	Using methods to assess additive interaction even when utilizing ratio effect estimates
Block/set regression	Sequentially adding sets of variables to regression models using forward selection to assess the impact of adding intersectional position to models that include each individual social position
Raw data[1]	Presenting raw data such as frequency of the outcome in each intersectional group

[1] This approach is not noted in Guan et al. but was added to the list of categories because it was employed by some of the studies included in this review
